# Structural comparison of metabolic networks in selected single cell organisms

**DOI:** 10.1186/1471-2105-6-8

**Published:** 2005-01-14

**Authors:** Dongxiao Zhu, Zhaohui S Qin

**Affiliations:** 1 Bioinformatics Program, University of Michigan, Ann Arbor, MI 48109, USA; 2 Center for Statistical Genetics, Department of Biostatistics, University of Michigan, Ann Arbor, MI 48109, USA; 3 Department of Statistics, University of Michigan, Ann Arbor, MI 48109, USA

## Abstract

**Background:**

There has been tremendous interest in the study of biological network structure. An array of measurements has been conceived to assess the topological properties of these networks. In this study, we compared the metabolic network structures of eleven single cell organisms representing the three domains of life using these measurements, hoping to find out whether the intrinsic network design principle(s), reflected by these measurements, are different among species in the three domains of life.

**Results:**

Three groups of topological properties were used in this study: network indices, degree distribution measures and motif profile measure. All of which are higher-level topological properties except for the marginal degree distribution. Metabolic networks in Archaeal species are found to be different from those in *S. cerevisiae *and the six Bacterial species in almost all measured higher-level topological properties. Our findings also indicate that the metabolic network in Archaeal species is similar to the exponential random network.

**Conclusion:**

If these metabolic network properties of the organisms studied can be extended to other species in their respective domains (which is likely), then the design principle(s) of Archaea are fundamentally different from those of Bacteria and Eukaryote. Furthermore, the functional mechanisms of Archaeal metabolic networks revealed in this study differentiate significantly from those of Bacterial and Eukaryotic organisms, which warrant further investigation.

## Background

Classification of biological organisms is of fundamental importance to evolutionary studies. It is commonly believed that there are three domains of life: Archaea, Bacteria and Eukaryote. Currently, the most popular classification method is the so called "molecular approach", in which polymorphism information in DNA or protein sequence is exploited to assess the phylogenetic relationships among species [1,2]. To a large extent, this is a "local" approach since the choice of sequence for comparison greatly affects the final result, "lateral gene transfer" (LGT) and thus the resulting "genome chimerism" further complicates the situation [3]. A new "system" approach that takes "global" properties of each organism into consideration serves as a potential alternative to overcome this shortcoming. Indeed, recent advances in system biology and increasingly available genomic databases have made it possible to rebuild biological networks from genomic data and have offered opportunity for such a "system" approach [4].

Podani and co-workers [5] proposed classifying organisms based on two kinds of network indices: the Jaccard index, which measures proportions of common sets of nodes in two networks, and Goodman-Kruskal *γ *function, which measures the similarity between rankings of nodes in two networks. They studied metabolic and information network structures of 43 organisms using these two measures under the hypothesis that network structure and the network design principle(s) behind them contain phylogenetic information. Ma and Zeng [6] conducted a more extensive phylogenetic classification study on 82 fully sequenced organisms based on different cellular function systems (enzyme, reaction, and genes) at the genomic level. They constructed phylogenetic tree based on Jaccard index and Korbel's definition, and concluded that in general, the classification based on network indices are in good agreement with the one obtained by analyzing the 16S rRNA using molecular approach. These studies seem to support the notion that significant differences in the network design principle(s) exist among the three domains of life [7]. These differences may reflect on the different approaches that organisms take to organize their entire systems to serve their special needs in the environment they live during the evolutionary history. Motivated by these encouraging results, in this manuscript, we went on to conduct a thorough comparison of network structural properties which provide further and more compelling evidences that significant differences exist among the network design principle(s) in organisms from the three domains of life.

Restricted by the theoretical network structural studies, there are not many deterministic and informative topological measurements available [8-11]. The established measurements can be roughly divided into two categories: higher-level (global) properties and low-level (local) properties. The difference between the two is that one needs to know the whole network in order to calculate the higher-level property measures (e.g. average path length) while the low-level properties can be worked out locally (e.g. marginal degree of individual node) [9]. We use three groups of topological measurements (both low and higher-level) that address different aspects of the network structure. The first group contains network indices such as average clustering coefficient, average path length [12]. The second group is composed of degree distributions (both marginal and bivariate joint degree distributions) [8-11,13]. The third group is composed of network motif profiles that are recently shown to represent the network design principle(s) and global statistical properties of the network when aggregating together [14-16]. These measurements have been well studied in the network literatures, and are able to capture most aspects of network degree information.

Single cell model organisms such as *E. coli *and *S. cerevisiae *have been studied intensively in biochemistry, cell biology and genetics; hence the rebuilt networks in those organisms present the best chance to approximate the true underlying network. Moreover, single cell organisms are less likely to have experienced the Whole Genome Duplication (WGD), which might drastically change the network structure [17,18]. As a result, we selected eleven single cell organisms to study their network structural properties: one Eukaryote: *S. cerevisiae*; six Bacteria: *E. coli*, *V. cholerae*, *R. solanacearum*, *B. subtilis*, *L. lactis*, *S. coelicolor*; and four Archaea: *S. solfataricus*, *S. tokodaii*, *M. acetivorans*, *T. acidophilum*.

There are three main types of intracellular networks: the protein-protein interaction network, the transcriptional regulation network and the metabolic network. The first two are rebuilt by using high throughput techniques such as yeast two-hybrid system, *in vivo *pull down assay or DNA microarray, which are subject to high uncertainties, and the resulting networks may not be good approximation to biological complexity [19-22]. On the other hand, the metabolic network is derived from metabolic pathways, many of which are inferred from biochemical experiment-defined stoichiometries of many reactions [23]. It is well known that central pathways contain "hub nodes" of the whole metabolic network [24,25] and are also main building blocks of the so-called Giant Strongly Connected Component (GSCC) and Giant Weakly Connected Components (GWCC) [26]. The former is defined as the largest cluster of nodes within which any pair of nodes is mutually reachable from each other, and the latter is defined as the largest cluster of nodes within which each pair of nodes is connected in the underlying undirected graph [10]. Therefore, our high confidence in the structure of GSCC and GWCC, based on experimentally verified pathways, guarantees high confidence in whole network structure. The long history of biochemical studies of enzymes ensures relatively low false positive and low false negative rates of connections. Therefore, we decided to use metabolic networks in single cell organisms to compare network topological properties in the three domains of life.

## Results

In constructing metabolic networks, Ma and Zeng [28] argued that connections through "current metabolites", which is referred to as cofactors in biochemistry such as ATP, ADP, H_2_O, should be removed from metabolic networks. We followed their suggestions by removing such "current metabolites" before conducting the following analysis.

### Group I measures: network indices

**Figure 1:**
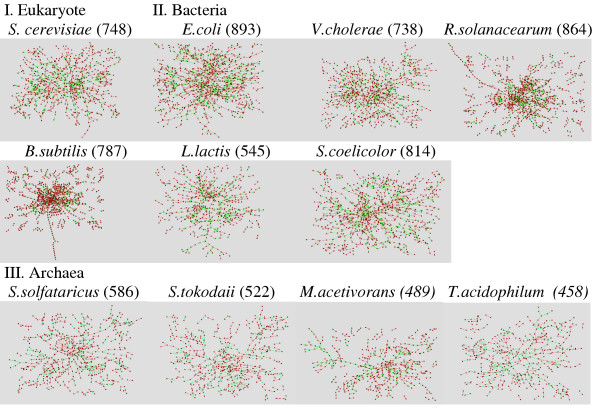
**Visualizations of metabolic networks in the eleven organisms. **In each graph, green lines represent arc and red lines represent edge. The numbers of distinct metabolites that are involved in at least one reaction are noted. All graphs are drawn with Pajek [33] using the layout optimization algorithm Kamada-Kawai.

Before checking different types of network topological measurements, we visually compared different metabolic networks (Fig. 1). Metabolic networks in *S. cerevisiae *and the six Bacterial species appear much more heterogeneous than Archaeal metabolic networks. It is well known that the so-called exponential random network (marginal degree distribution follows a Poisson distribution, see Methods for details) appears homogeneous while scale-free network (marginal degree distribution follows a power-law distribution, see Methods for details) appears more heterogeneous and modular [9].

**Figure 2:**
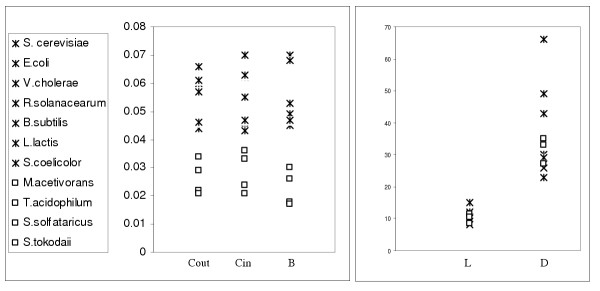
Five network indices (Clustering Coefficients (C_out_, C_in_), Betweenness (B), Average Path Length (L) and Diameter (D)) of the metabolic networks in the eleven organisms.

Calculations of the two classic network indices, average clustering coefficient and average betweenness (see Methods for definition) also indicate that the metabolic networks in *S. cerevisiae *andthe six Bacterial species are more clustered and modular than those in the four Archaeal species (Table 1, Fig. 2). From Table 1 and Fig. 2, it is evident that the Clustering Coefficient (C) and Betweenness (B) did a better job in separating Archaeal species from non-Archaeal species than Average Path Length (L) and Diameter (D). Note that since we removed connections through "current metabolites" when constructing metabolic networks, our average path lengths are much longer than those reported in Jeong et al. [25] but similar to those reported in Ma and Zeng [28].

**Figure 3:**
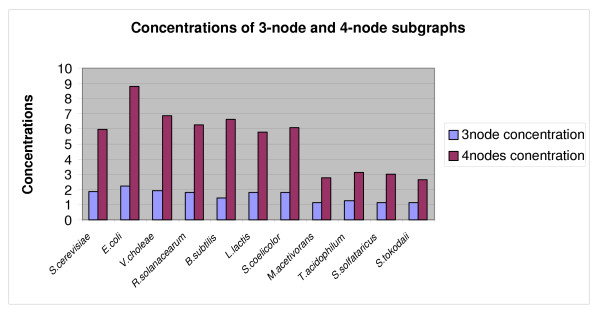
Concentrations (number of appearances divided by number of nodes with edges/arcs) of three-node and four-node subgraphs.

To avoid the confounding effects stemming from different network sizes, we calculated the so-called concentrations (number of appearances of subgraphs divided by the number of nodes with edges or arcs (directed edges), see Methods for details) of three-node subgraphs and four-node subgraphs. The concentration of subgraphs is an objective measure of the extent of clustering and modularity of the network [8,9]. It is observed that the concentrations of subgraphs in *S. cerevisiae *and the six Bacterial metabolic networks are much higher than those in Archaeal metabolic networks (Fig. 3).

### Group II measures: degree distributions

#### Marginal degree distributions

**Figure 4:**
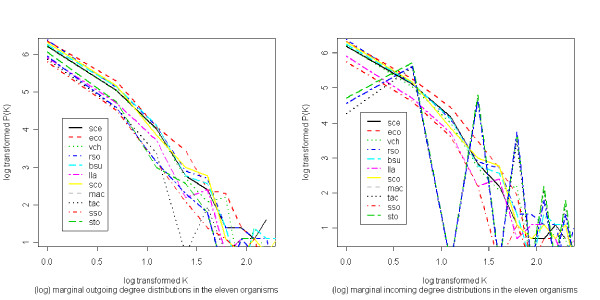
(A) Log transformed marginal outgoing degree distributions (B) Log transformed marginal incoming degree distributions in the eleven organisms

Recently, a variety of real-life networks are found to share the "scale-free" property, i.e. the marginal degree distribution follows a power-law distribution [25,29-31]. Our analysis demonstrates that the outgoing and incoming marginal degree distributions in metabolic networks also follow the power-law distribution. A simple linear model fits the log-transformed data well (except for the incoming degree distributions for most of the Archaea) which indicates that in general, the power-law model is appropriate to capture the structure of degree data (Fig. 4). Parameters were estimated using the Least Square method. The results together with goodness of fit measure *R*^2 ^and 95% individual confidence intervals are summarized in Table 2 and Table 3. The estimated power-law index *γ *is around -0.3 in all cases and the estimated log-transformed scaling parameter *α *ranges within 2.0 to 2.5. These indicate that marginal degree distribution, which is a low-level (local) topological property measure, although showed some distinction, is not enough to effectively differentiate networks from different domains. Overall, metabolic networks in most of the species we studied seem to follow the power-law distributions and thus are "scale-free". The fact that the incoming degree distributions of most Archaeal species we studied do not follow power-law well (Fig. 4B) suggests that networks in Archaeal species tend to be less "scale-free" and more "random-like" compared to those of the non-Archaeal species.

As we have shown, marginal degree distribution alone does not reveal the fundamental network structural differences between the Archaeal species and the non-Archaeal species. Simulation studies have shown that randomized networks preserving marginal degree distribution can be quite different in terms of global (higher level) topological properties such as average clustering coefficient [9]. In metabolic networks, we are unable to determine the preferred types of reactions based on just marginal substrate or product degree distributions. Since the metabolic network is rebuilt from chemical reactions, joint behavior of substrate and product in reactions should be more informative than disjoint behavior of metabolites. Therefore, we calculate the joint degree distributions hoping to gain more insight into the network organization.

#### Joint degree distributions

Joint degree distribution measures and describes correlation between connectivities of neighboring nodes. *N*(*K*_0_, *K*_1_) is defined as the number of edges connecting nodes of connectivity *K*_0 _to those of connectivity *K*_1_. For metabolic networks, which are directed, *N*(*K*_*out*_, *K*_*in*_) is used to measure the number of arches where substrate (node) with out-connectivity *K*_*out *_transforms to product with in-connectivity *K*_*in*_. This quantity reflects intrinsic properties of the network and can be used to distinguish different types of networks. For instance, we can test whether *N*(*K*_*out*_, *K*_*in*_) of a particular network differs significantly from that of the random network. To be specific, we calculate , where (*K*_*out*_, *K*_*in*_) represents the mean of random variable *N*(*K*_*out*_, *K*_*in*_) in a large number (say, 1000) of random networks simulated by an edge-rewiring algorithm proposed by Maslov and Sneppen [13], (*K*_*out*_, *K*_*in*_) denotes the estimated standard deviation of *N*(*K*_*out*_, *K*_*in*_). The *p*-value can then be obtained by compare *Z *to a standard normal distribution. Comparing with "properly" randomized network ensembles allows us to concentrate on those statistically significant patterns of the complex network that are likely to reflect the design principle(s) [13].

**Figure 5:**
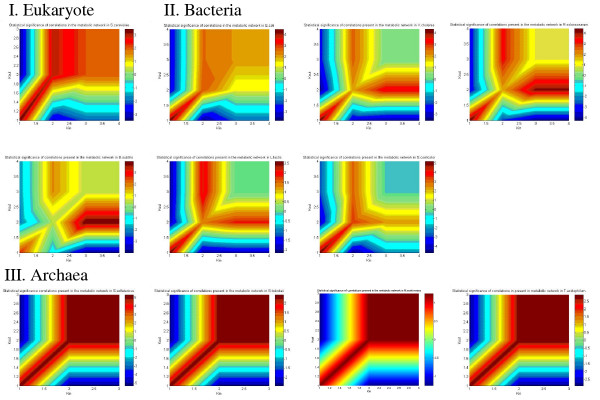
**Statistical significance of correlation (Z-scores) present in the metabolic networks. **To improve statistics, the connectivities in all eleven panels of this figure were logarithmically binned into two bins per decade. Statistically significant correlation profiles are generated using the Matlab program developed by Maslov and Sneppen [13].

We calculated statistically significant correlation profiles (Z-score profiles, see Methods for details) for the metabolic network in each organism (Fig. 5). The Z-score profiles of the four Archaeal species are similar to each other but quite different from those in *S. cerevisiae *and the six Bacterial species. Although the dark red regions of the Z-score profiles in Archaeal species are quite different in scale, they all seem to differ significantly from the random network preserving the corresponding marginal degree distribution in a similar way (*p*-value < 0.1). Looking into the correlation profiles more carefully, we found that the number of statistically significant positive (*K*_*out*_, *K*_*in*_) increases in the order of *S. cerevisiae*, the six Bacterial species and the four Archaeal species. The significant Z-score of certain observation *N*(*K*_out_, *K*_*in*_) implies that the chemical reaction between substrates with out-degree *K*_*out *_and products with in-degree *K*_*in *_are statistically significant. We define substrates whose *K*_*out *_>= *2 *or products whose *K*_*in *_>= *2 *as versatile metabolites. Thus, the above trend implies that the preference to employ reactions involving versatile metabolites increases in the order of *S. cerevisiae*, the six Bacterial species and the four Archaeal species. Correspondingly, the variety of metabolites decreases in the above order and so does the number of distinct enzymes or variety of enzymes because of the high specific binding of metabolites and enzyme. This is consistent with the biological facts that *S. cerevisiae *(Eukaryote) encodes a greater variety of enzymes than Bacterial and Archaeal species.

### Group III measure: Network Motif

The network motif is defined to be recurring and non-random building blocks of the network [14,15]. Just like sequence motif, which is an over-represented and biologically meaningful DNA or protein sub-sequence, network motif is an over-represented and biologically meaningful subgraph.

Network motif has been shown to be informative of network design principle(s) and network structure. It was found that over 80% of the nodes in the *E. coli *transcription regulation network are covered by network motifs [14]. Dobrin et al. [16] recently discovered that in the *E. coli *transcriptional regulatory network, "individual motifs aggregate into homologous motif clusters and a supercluster forming the backbone of the network and play a central role in defining its global topological organization." More importantly, network motifs capture the information that is likely to be missed by the correlation profiles because motif actually describes the number of appearances of certain configurations of multiple nodes, and therefore nicely complement with the correlation profiles [9]. One might argue that there are certain amount of overlaps between the information they capture but the motif profile does not capture the degree information of the connecting nodes, which may be the most powerful feature of the correlation profiles.

**Figure 6:**
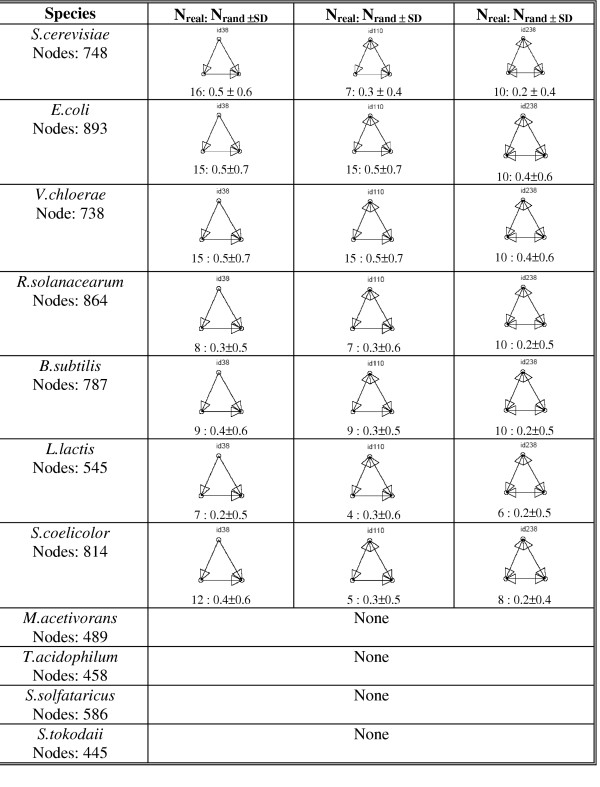
**Three-node motifs found in the metabolic networks in different species. **The number of connecting nodes for each network is shown. For each motif, the numbers of appearances in real networks (*N*_*real*_) and in randomized networks (*N*_*rand *_± *SD*, all values rounded) are shown. The *p*-values of all motifs are less than 0.01, as determined by comparing to 1000 randomized networks. Each motif occurs at least four times in one network. Motifs were detected and generated using program developed by Milo et al. (2002) and the motif dictionary therein [15].

We searched for all of the 13 three-node subgraphs and all of the 199 four-node subgraphs in the metabolic networks of eleven species. The results showed that the three-node motif profiles found in *S. cerevisiae *and the six Bacterial species are identical while there is no three-node motif found in any of the four Archaeal networks (Fig. 6). Also there is no common four-node motif shared by Archaeal species and *S. cerevisiae*/Bacterial species while two four-node motifs (id4702, id4950) are shared by the latter (Additional file 1). Among all the 13 possible three-node subgraphs, six of them have one pair of nodes not directly connected. Abundance of such subgraphs will lower the extent of clustering and modularity of the network. As expected, we found that all three-node motifs identified in *S. cerevisiae *and the six Bacterial species form triangles (Fig. 6). It may explain our main finding that metabolic networks in non-Archaeal species are more clustered and modular than those in Archaeal species.

## Discussion

Based on our comparison of network structural properties beyond network indices, we were able to gain more insight into the structural differences across the three domains of life. Having shown that the metabolic network is "scale-free", we further showed that metabolic networks in the four Archaeal species are closer to "exponential random network" [9:Ch2, [11]] than those in *S. cerevisiae *and the six Bacterial species. The reasons are the following:

First, the Archaeal metabolic networks are visually more homogeneous among themselves compared to their counterparts in the non-Archaeal species. In random networks, any pair of nodes is equally likely to be connected. The network topology should look homogeneous given that the size of network is large enough. The "scale-free" network, on the other hand, features a highly modular and heterogeneous topology since the marginal degree is power-law distributed [8,9]. Moreover, the marginal degree distributions of the metabolic networks in non-Archaeal species fit the power-law model better than Archaeal species (Table 2 and Table 3).

Second, the average clustering coefficient and average betweenness of Archaeal metabolic networks are much smaller than those in *S. cerevisiae *and the six Bacterial species. The same is true for the concentrations of three-node and four-node subgraphs. As pointed out by Watts and Strogatz, real-life networks show strong clustering or network transitivity while exponential random network does not [12].

Third, there is no three-node motif and fewer four-node motifs found in Archaeal metabolic networks compared to non-Archaeal metabolic networks. In particular, the ubiquitous feed-forward loop (FFL) motif found in networks from biology (including metabolic networks in *S. cerevisiae *and the six Bacteria species in this study) to neurology and engineering fields was not found in any of the four Archaeal metabolic networks (Fig. 6). Since motifs are statistically significant subgraphs compared to "properly" randomized network ensembles, no motif or fewer than usual motifs found in a real-life network indicates that the network structure is closer to that of a random network. It has been shown by Milo et al. [15] that concentration of FFL motif is insensitive to the network size within *E. coli *transcription regulation network, but diminishes to zero in increasingly larger random networks. This also supports that Archaeal metabolic networks are closer to randomized network ensembles than other real-life networks.

The metabolic networks in Archaea are both "random-like" and "scale-free", which might exert profound influences on their adaptability to the hostile environment. Archaeal species are typically restricted to marginal habitats such as hot springs or areas of low oxygen concentration and can assimilate different kinds of inorganic carbon and nitrogen sources. Indeed, the chemical structure and component of the macromolecules such as protein and lipid make significant contributions to the organism's adaptability to the environment. The seemingly *ad hoc *network organization (both "random-like" and "scale-free") in Archaeal species might also enabled them to survive in those extreme physiological conditions. Archaeal species might employ some biologically significant subgraphs (rather than statistically significant motifs) which can not be detected by current motif searching algorithm [15]. This makes the Archaeal metabolic networks appear random in statistical sense (not statistically significantly different from random networks) but not in biological sense.

Our comparison results showed that many network structural properties measured in Archaeal species are different from those of non-Archaeal species. However, the hidden anthropomorphic factors might account for some of the differences observed. Specifically, the drastic differences of topological profiles between the metabolic networks of Archaeal species and non-Archaeal species may be partially explained by the fact that significantly less extensive metabolic pathway studies have been conducted in Archaeal species [32]. Robustness of topological profiles against random perturbations can alleviate the impact to a certain extent but is unable to eradicate it [9].

## Conclusions

Our network analysis results showed that in most of higher-level (global) topological properties measured, metabolic networks in the four Archaeal species are similar to each other but significantly different from those in *S. cerevisiae *and the six Bacterial species. This provides further evidence that the metabolic network structures and consequently the design principle(s) in the four Archaeal species are very different from those in *S. cerevisiae *(Eukaryote) and the six Bacterial species. Our finding that the metabolic networks in Archaeal species possess many properties of the exponential random network begs for better understanding of the design principle(s) in biological networks, which may be revealed by further systematic analyses. For example, locate and align conservative pathways such as glycosis between *E. coli *or *S. cerevisiae *and Archaeal species to understand the functional mechanisms of Archaeal metabolic networks.

## Methods

### Data source

Chemical reaction data was obtained from metabolic database in Ma and Zeng [28], which consists of five related tables: *reaction*, *enzyme*, *react*, *connect *and *organism*. We compiled a new table from this database excluding any inconsistent or redundant connections between metabolites (details below). SQL was used to query the database.

### Identify and remove inconsistency

Inconsistent connections refer to pairs of metabolites that have conflicting reversibility annotation. It is caused by the fact that a pair of metabolites can be in more than one reaction and the reversibility of these reactions can be different. For example, NAD^+ ^and Nicotinamide is a pair of metabolites in two reactions: 1) **NAD**^+ ^+ L-Arginine = **Nicotinamide **+ N_2 _(ADP-D-ribosyl)-L-arginine 2) **NAD**^+ ^+ H_2_O ->**Nicotinamide **+ ADPribose. (Note that here the role of NAD^+ ^is NOT "current" metabolite, and hence connections established through it should NOT be removed). Reaction 1 is a reversible reaction while reaction 2 is not. We annotated an edge between the two metabolites as long as there was at least one reversible reaction that both of them were involved. For example, the type of connection between **NAD**^+ ^and **Nicotinamide **is edge (undirected connection). This step could be summarized as "edge ← edge + arc".

### Identify and remove redundancy

There are also numerous redundant connections where the same pair of metabolites switch their roles between substrate and product in two or more different irreversible reactions. For example: 1) UDPglucose + **N-Acylsphingosine **= UDP + **Glucosylceramide **2) **Glucosylceramide **+ H_2_O = D-Glucose + **N-Acylsphingosine**. (**N-Acylsphingosine **and **Glucosylceramide **is a pair of metabolites that switch their roles in two irreversible reactions). In case of redundancy, we annotated an edge between the pair of metabolites rather than the two arcs because they could be converted to each other through two reactions. This step could be summarized as "edge ← arc + arc".

### Definitions of some network topological measurements

#### Clustering coefficient (C)

We define two kinds of clustering coefficients for each node in the directed metabolic networks, i.e. *C*_*in *_and *C*_*out*_. *C*_*in *_measures the average clustering coefficient of the node representing the product that can be generated from its first-order "nearest neighbors" through chemical reactions. *C*_*out *_measures the average clustering coefficient of the node that generate its first-order "nearest neighbors" through chemical reactions. The larger the coefficients, the more clustered and modular the network appears to be.

#### Betweenness (B)

The betweenness for any node *n*_*i *_in the network is defined as , where *g*_*jk *_is the number of shortest paths between node *j *and node *k*. *g*_*jk*_(*n*_*i*_) is the number of shortest path between node *j *and node *k *containing node *n*_*i*_, *g *is the total number of nodes with edges/arcs. *C*_*B*_(*n*_*i*_) needs to be multiplied by two in the case of directed network [27]. The average betweenness is defined as: . Higher value of betweenness indicates the network is more clustered and modular.

#### Average path length (L)

Watts and Strogatz [12] defined the average path length as , where *d*(*j*, *k*) is the shortest path length between node *j *and node *k *(distance), *V *represents the set of all nodes with edges/arcs of the graph, and *g *is the number of nodes with edges/arcs.

#### Diameter (D)

The diameter of the directed graph *G *is the longest geodesic between any pairs of nodes. The geodesic is the shortest path between a pair of nodes. Pajek [33] was used to calculate the average betweenness, average path length and diameter.

### Concentration of subgraphs (S)

Wasserman and Katherine [27] defined the subgraph as follows: A graph *G*_*s *_is a subgraph of *G *if the set of nodes of *G*_*s *_is a subset of the set of nodes of *G*, and the set of lines in *G*_*s *_is a subset of the lines in the graph *G*. Let *M *be the number of subgraphs, and *N *be the number of nodes with edges or arcs. Then the "concentration of subgraph" is defined as *C *= *M/N*. A high value of *C *indicates the network is more clustered and modular. Mfinder1.1 [15] was used to calculate both *M *and *N*.

### Marginal degree distribution calculations

The marginal degree distribution of each network is calculated from the Boolean adjacency matrix *A*, a matrix of 0 or 1. Zero means there is no connection between nodes, and 1 the opposite. The outgoing degree of the node *i*, *k*_*out*(*i*) _is defined as , where . The incoming degree of the node *i*, *k*_*in*(*i*) _is defined as .

### Simple regression analyses of marginal degree distributions

The power-law degree model was first log transformed into linear model, i.e. log *P*(*K*_*i*_) = *γ *log(*K*_*i*_) + log(*α*) + *ε*_*i *_(*i *= *1,2,...,n*), *γ *and *α *are parameters, *ε*_*i *_is the residual. *K*_*i *_is the degree and *P*(*K*_*i*_) is the corresponding probability. Based on the fitted linear model, we made statistical inference including parameter estimation and individual confidence intervals on the estimates using the Least Square method.

### Correlation profile calculations

Statistically significant correlation profiles were calculated using Matlab code downloaded from Dr. Maslov's website [34]. The adjacency matrix of the network is the input.

### Motif profiles calculations

According to Milo et al.[15] , a subgraph is referred to as a motif if the following criteria are met: 1) Its empirical *p*-value is smaller than a pre-specified threshold, e.g. 0.01. 2) The number of appearances in real networks with distinct sets of nodes is larger than another pre-specified cut-off value, e.g. 4. 3) The number of appearances in real networks is significantly larger than that in randomized networks, i.e. . *N*_*real *_and *N*_*rand *_represent the number of certain subgraphs detected in real-life network and randomized networks, respectively. This is to avoid the situation where some common subgraphs are detected as motifs that have only slight differences in *N*_*real *_and *N*_*rand *_but have a narrow spread of distribution in randomized networks [14,15]. Motif profiles are generated using the Mfinder program. This program and the motif dictionary were downloaded from Dr. Uri Alon group's website [35].

## Authors' contributions

DZ and ZSQ conceived and designed the study; DZ wrote the computer code, analyzed the data and draft the manuscript. Both authors read and approved the final manuscript.

## Supplementary Material

Additional File 1 **Four-node motifs found in the metabolic networks in different species. **The number of connecting nodes for each network is shown. For each motif, the numbers of appearances in real networks (*N*_*real*_) and in randomized networks (*N*_*rand *_± *SD*, all values rounded) are shown. The *p*-values of all motifs are less than 0.01, as determined by comparing to 1000 randomized networks. Each motif occurs at least four times in one network. Other restrictions apply. Motifs were detected and generated using program found in Milo et al. [15] and the motif dictionary therein. Click here for file

## Figures and Tables

**Table 1:** Descriptive statistics of metabolic networks in the eleven organisms.

**DOMAIN, KINGDOM AND PHYLUM**			**ORGANISM**	**NUM NODES**	**NUM EDGES**	**SINGLE EDGES**	**MUTUAL EDGES**	**C_OUT_**	**C_IN_**	**B**	**L**	**D**
Eukarya			*S. cerevisiae*	748	1072	396	338	0.066	0.062	0.053	12.147	49
Bacteria	Proteobacteria	gamma	*E. coli*	893	1365	459	453	0.060	0.070	0.070	9.281	30
			*V. cholerae*	738	1076	370	353	0.057	0.055	0.045	8.236	23
		beta	*R. solanacearum*	864	1238	406	416	0.044	0.044	0.049	10.358	43
	Firmicutes	Bacillales	*B. subtilis*	787	1151	401	375	0.061	0.063	0.047	10.020	29
		Lactobacillales	*L. lactis*	545	778	280	249	0.044	0.043	0.068	9.277	27
	Actinobacteria		*S. coelicolor*	814	1154	406	374	0.046	0.047	0.047	15.062	66
Archaea	Euryarchaeota		*M. acetivorans*	489	633	209	212	0.029	0.033	0.026	11.350	35
			*T. acidophilum*	458	593	197	198	0.034	0.036	0.030	10.597	33
	Crenarchaeota		*S. solfataricus*	586	730	256	237	0.022	0.021	0.018	8.053	26
			*S. tokodaii*	522	651	229	211	0.021	0.024	0.017	8.424	27

The column marked "Num nodes" lists the number of metabolites that are involved in at least one chemical reaction in the organism. The column marked "Num edges" lists the number of all directed chemical reactions in the organism. Note that this number consists of two parts: The number of irreversible reactions, i.e. "Single edges"; and the number of reversible reactions, i.e. "Mutual edges", where "Num edges" = "Single edges" + 2 × "Mutual edges". The column marked "*C*_*out*_" lists the average clustering coefficient calculated from the nearest neighbors in out-component. The column marked "*C*_*in*_" lists the average clustering coefficient calculated from the nearest neighbors in in-component. Column marked "B" lists the average betweenness of the network, the column "L" lists average path length of the network and column marked "D" lists diameter of the network.

**Table 2:** Parameter estimates of *γ *and log*α *in the outgoing degree distribution model.

	**R^2^**	***γ*, 95% C.I.**	**log*α*, 95% C.I.**
***S. cerevisiae ***(748)	0.96	-0.39, [-0.46, -0.31]	2.53, [2.29, 2.78]
***E. coli ***(893)	0.92	-0.36, [-0.43, -0.28]	2.51, [2.29. 2.74]
***V. cholerae ***(738)	0.91	-0.36, [-0.44, -0.28]	2.45, [2.22, 2.68]
***R. solanacearum ***(864)	0.96	-0.37, [-0.43, -0.31]	2.50, [2.32, 2.68]
***B. subtilis ***(787)	0.92	-0.36, [-0.44, -0.28]	2.46, [2.23, 2.68]
***L. lactis ***(545)	0.95	-0.38, [-0.45, -0.31]	2.39, [2.20, 2.58]
***S. coelicolor ***(814)	0.95	-0.36, [-0.43, 0.30]	2.47, [2.29, 2.65]
***S. solfataricus ***(586)	0.92	-0.33, [-0.43, -0.23]	2.17, [1.86, 2.49]
***S. tokodaii ***(445)	0.94	-0.34, [-0.42, -0.25]	2.15, [1.88, 2.42]
***T. acidophilum ***(458)	0.97	-0.37, [-0.44, -0.31]	2.25, [2.05, 2.45]
***M. acetivorans ***(489)	0.86	-0.33, [-0.46, -0.20]	2.13, [1.73, 2.53]

Model: log *P*(*K*_*i*_) = *γ *log(*K*_*i*_) + log(*α*) + *ε*_*i *_(*i *= 1,2,...,*n*). Parameters are estimated using Least Square Method.

**Table 3:** Parameter estimates of *α *and *γ *in the incoming degree distribution model.

	**R^2^**	***γ*, 95% C.I.**	**log*α*, 95% C.I.**
***S. cerevisiae ***(748)	0.95	-0.35, [-0.42, -0.29]	2.38, [2.18, 2.59]
***E. coli ***(893)	0.90	-0.35, [-0.42, -0.28]	2.50, [2.29, 2.70]
***V. cholerae ***(738)	0.91	-0.36, [-0.44,-0.28]	2.45, [2.22, 2.68]
***R. solanacearum ***(864)	0.96	-0.37, [-0.42, -0.31]	2.50, [2.32, 2.68]
***B. subtilis ***(787)	0.92	-0.36, [-0.43, -0.28]	2.46, [2.23, 2.68]
***L. lactis ***(545)	0.95	-0.38, [-0.45, -0.31]	2.40, [2.20, 2.58]
***S. coelicolor ***(814)	0.95	-0.36, [-0.43, -0.30]	2.47, [2.29, 2.65]
***S. solfataricus ***(586)	0.45	-0.24, [-0.41, -0.07]	2.21, [1.76, 2.68]
***S. tokodaii ***(445)	0.46	-0.25, [-0.42, -0.08]	2.21, [1.76, 2.65]
***T. acidophilum ***(458)	0.89	-0.30, [-0.41, -0.20]	2.00, [1.69, 2.32]
***M. acetivorans ***(489)	0.46	-0.25, [-0.43, -0.08]	2.20, [1.75, 2.65]

Model: log *P*(*K*_*i*_) = *γ *log(*K*_*i*_) + log(*α*) + *ε*_*i *_(*i *= 1,2,...,*n*). Parameters are estimated using Least Square Method.
