# Supplementary material for: Structural comparison of metabolic networks in selected single cell organisms
Source: BMC Bioinformatics. 2005 Jan 14;6:8. doi: 10.1186/1471-2105-6-8 (PMC549204; doi:10.1186/1471-2105-6-8)
Supplement: Additional File 1 — Four-node motifs found in the metabolic networks in different species. The number of connecting nodes for each network is shown. For each motif, the numbers of appearances in real networks (Nreal) and in randomized networks (Nrand ± SD, all values rounded) are shown. The p-values of all motifs are less than 0.01, as determined by comparing to 1000 randomized networks. Each motif occurs at least four times in one network. Other restrictions apply. Motifs were detected and generated using program found in Milo et al. [15] and the motif dictionary therein. [file 1471-2105-6-8-S1.doc]

| Species | **N_real_: N_rand ±SD_** | **N_real_: N_rand ± SD_** | **N_real_: N_rand ± SD_** |
| --- | --- | --- | --- |
| S.cerevisiae | 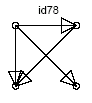 | 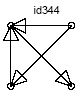 | 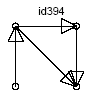 |
|  | 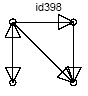 | 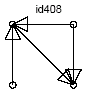 | 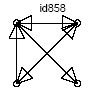 |
|  | 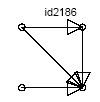 | 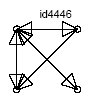 | 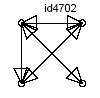 |
|  | 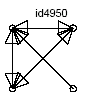 | 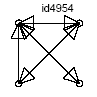 | 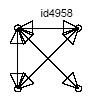 |
| E.coli | 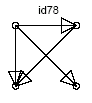 | 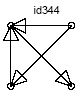 | 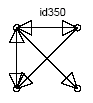 |
|  | 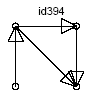 | 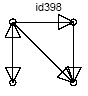 | 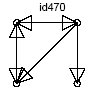 |
|  | 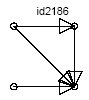 | 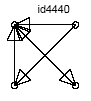 | 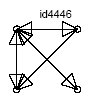 |
|  | 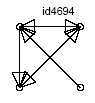 | 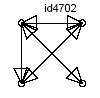 | 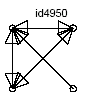 |
|  | 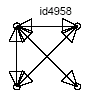 | 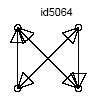 | 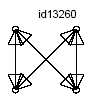 |
| *V.cholerae* | 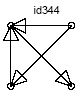 | 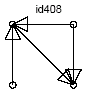 | 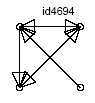 |
|  | 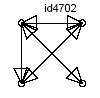 | 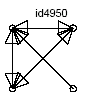 | 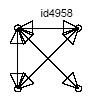 |
|  | 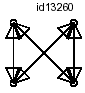 |  |  |
| *R.solanacearum* | 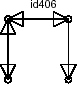 | 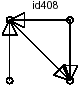 | 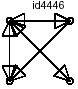 |
|  | 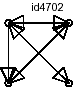 | 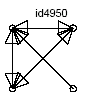 |  |
| *B.subtilis* | 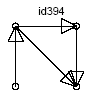 | 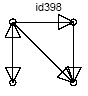 | 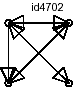 |
|  | 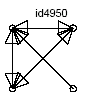 | 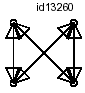 |  |
| *L.lactis* | 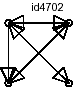 | 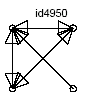 |  |
| S.solfataricus | 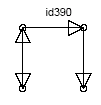 | 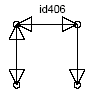 | 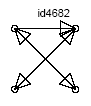 |
| S.tokodaii | 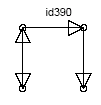 | 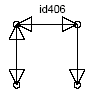 |  |
| M.acetivorans | 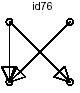 |  |  |
| *T.acidophilum* | None |  |  |
